# Prevalence, Reasons, and Determinants of Discharge Against Medical Advice Among Hospitalized Pediatric Patients at the University of Gondar Comprehensive Specialized Hospital: A Prospective Observational Study

**DOI:** 10.1002/hsr2.72979

**Published:** 2026-08-17

**Authors:** Tesfaye Shumet Mekonnen, Demewoz Fikir

**Affiliations:** ^1^ Department of Public Health College of Medicine and Health Sciences, Debre Markos University Debre Markos Ethiopia; ^2^ Department of Pediatrics and Child Health School of Medicine, College of Medicine and Health Sciences, University of Gondar Gondar Ethiopia

**Keywords:** determinants, discharge against medical advice, Ethiopia, pediatrics, prevalence

## Abstract

**Background and Aims:**

Discharge against medical advice (DAMA) is a major challenge in pediatric care, leading to poor treatment outcomes, increased readmissions, and preventable mortality. Evidence on the prevalence and determinants of pediatric DAMA in Ethiopia is limited. This study aimed to assess the prevalence, reasons, and factors associated with DAMA among pediatric patients admitted to the University of Gondar Comprehensive Specialized Hospital (UGCSH), Northwest Ethiopia.

**Methods:**

A hospital‐based prospective observational study was conducted from December 1, 2024, to September 30, 2025. A total of 422 participants were selected using systematic random sampling. Data were collected through caregiver interviews and medical record review using a structured questionnaire, with participants enrolled at admission and discharge outcomes were assessed at hospital discharge. Descriptive statistics were used. Variables with *p* < 0.25 in bivariable analyses were included in multivariable logistic regression, and *p* < 0.05 was considered statistically significant.

**Results:**

Of 422 pediatric patients, 42 (9.7%; 95% CI: 7.1–12.8%) were discharged against medical advice. DAMA was more common among males (63.4%) and children aged ≥ 5 years (15.4%). DAMA was most frequent in the pediatric inpatient ward (36.5%). Financial constraints, lack of clinical improvement, and perceived recovery were the main reasons. In multivariable analysis, maternal education was significantly associated with DAMA. Children whose mothers had secondary education had 68% lower odds of DAMA (AOR = 0.32; 95% CI: 0.11–0.99; *p* = 0.04), while children whose mothers had tertiary education had 81% lower odds (AOR = 0.19; 95% CI: 0.04–0.84; *p* = 0.03) compared with children of mothers with no formal education.

**Conclusion:**

Nearly one in ten hospitalized children were discharged against medical advice. Maternal education was significantly associated with DAMA, suggesting that socioeconomic factors and caregiver knowledge may play an important role in hospital discharge decisions. Interventions such as caregiver education and strengthening hospital services may help reduce DAMA and improve pediatric outcomes.

**Abbreviations**
AOR: Adjusted odds ratio
CI: confidence interval
COR: Crude Odds Ratio
DAMA: discharge against medical advice
IQR: interquartile range
NICU: neonatal intensive care unit
PICU: pediatric intensive care unit
PNA: perinatal asphyxia
SPSS: Statistical Package for the Social Sciences
STROBE: Strengthening the Reporting of Observational Studies in Epidemiology
UGCSH: University of Gondar Comprehensive Specialized Hospital
VIF: variance inflation factor

## 1 Introduction

Discharge against medical advice (DAMA) is when a patient leaves a healthcare facility before the treating clinician deems it appropriate [1, 2]. This phenomenon presents significant clinical, ethical, and legal challenges for both healthcare providers and institutions, as it disrupts continuity of care and exposes patients to avoidable health risks [3, 4]. Globally, thousands of patients leave hospitals prematurely each year, with studies consistently reporting higher rates of DAMA in low‐ and middle‐income countries compared with high‐income settings [5, 6].

Multiple factors contribute to DAMA, including financial constraints, dissatisfaction with services, perceived improvement in illness, mistrust of healthcare providers, and preference for traditional or alternative therapies [1, 7]. Effective communication and patient satisfaction are essential components of high‐quality care; when these are compromised, cooperation and treatment adherence decline. Additional personal and contextual factors, such as socioeconomic status, limited health literacy, parental beliefs, cultural practices, and reliance on traditional healers, also influence decisions to discontinue treatment prematurely [8, 9].

In pediatric care, DAMA has particularly serious consequences, as decisions are usually made by parents or guardians for minors [10, 11]. Premature discharge can cause clinical deterioration, long‐term complications, higher morbidity, and even preventable death. It also leads to increased readmission rates and greater healthcare costs [12]. Parental refusal of necessary medical treatment may raise concerns about child neglect, especially when such choices threaten the child's well‐being. DAMA is also upsetting for healthcare professionals, who may see these decisions as a failure of communication or trust [10, 13].

Studies show that the prevalence of pediatric DAMA varies widely, ranging from 1.2% to 31.7% across healthcare facilities [12, 13, 14]. Evidence indicates that children discharged against medical advice are more likely to experience treatment failure and require unscheduled readmission [15]. In pediatric settings, readmission prevalence after DAMA has been reported to range from approximately 20% to 28% within 30 days, underscoring the risk of incomplete treatment [7]. While older children and adolescents may sometimes participate in decisions regarding their discharge, the ultimate decision typically remains with parents or guardians [16]. When DAMA poses an immediate threat to the child's health, child protection issues may need to be considered.

In Ethiopia, DAMA represents an important yet underexplored challenge, particularly in settings where health insurance coverage is limited and out‐of‐pocket costs remain high. Anecdotal evidence suggests that financial hardship, low parental education, and limited understanding of disease severity contribute significantly to DAMA among hospitalized children [1, 17]. However, empirical data on the magnitude and determinants of pediatric DAMA in Ethiopia remain scarce.

Given the potential consequences of DAMA for child health outcomes and the strain it places on the healthcare system, it is essential to investigate the factors influencing these decisions. To date, few empirical studies have examined pediatric DAMA in Ethiopia, and comprehensive evidence on its prevalence, reasons, and determinants remains limited [1, 17].

Therefore, this study aimed to assess the magnitude and associated factors of discharge against medical advice among pediatric patients admitted to the University of Gondar Comprehensive Specialized Hospital, Northwest Ethiopia.

## 2 Methods

### 2.1 Study Design and Setting

A hospital‐based prospective observational study was conducted from December 1, 2024, to September 30, 2025, among pediatric patients admitted to the University of Gondar Comprehensive Specialized Hospital (UGCSH) in Northwest Ethiopia. Eligible patients were enrolled at admission, and discharge outcomes were assessed at the time of hospital discharge.

The study was conducted at the University of Gondar Comprehensive Specialized Hospital (UGCSH), a tertiary teaching hospital and one of the oldest health institutions in Ethiopia. The hospital is located in Northwest Ethiopia, 727 km from Addis Ababa, in the Amhara Regional State (12.50° N, 37.24° E), at an altitude of approximately 2380 meters above sea level [11]. UGCSH serves more than one million people annually from the Central, North, and Western Gondar zones.

The hospital is a public tertiary care institution where the government partially subsidizes healthcare services. However, patients may still incur out‐of‐pocket expenses for certain medications, diagnostic tests, and procedures. At the study hospital, neonates admitted to the neonatal intensive care unit (NICU) and children admitted to the malnutrition ward receive all healthcare services free of charge. In Ethiopia, community‐based health insurance (CBHI) has also been implemented to reduce financial barriers to healthcare access among enrolled households.

The Department of Pediatrics and Child Health provides both inpatient and outpatient services through the pediatric emergency department, pediatric intensive care unit (PICU), neonatal intensive care unit (NICU), oncology ward, malnutrition ward, and general pediatric wards [18].

### 2.2 Study Population and Eligibility Criteria

The study population included all pediatric patients admitted to the pediatric wards of UGCSH during the study period. Pediatric patients aged < 18 years were eligible for inclusion. At UGCSH, adolescents aged 15–17 years are routinely admitted and managed within pediatric services; therefore, this age group was included. Children admitted without a parent or caregiver were excluded.

### 2.3 Sample Size Determination and Sampling

The sample size was determined using a single population proportion formula, assuming the prevalence of DAMA to be 50% (due to lack of previous studies in Ethiopia), a 5% margin of error, and a 95% confidence level. Using a *Z*‐value of 1.96, the calculated sample size was 384. After adding a 10% non‐response rate, the final sample size was 422.

**Figure 1:**
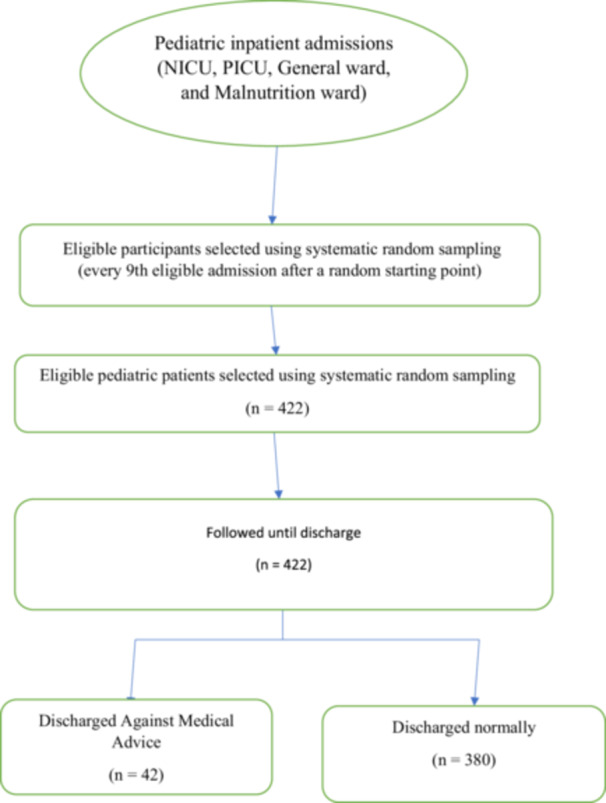
Flow diagram illustrating participant selection, enrollment, and classification of discharge outcomes among pediatric patients admitted to the University of Gondar Comprehensive Specialized Hospital, Northwest Ethiopia, December 2024–September 2025.

The pediatric inpatient units (NICU, PICU, general pediatric inpatient ward, and malnutrition ward) were considered strata. The required sample size was allocated across the pediatric inpatient units based on expected patient flow. The sampling interval (k) was calculated by dividing the estimated number of eligible pediatric admissions during the study period by the required sample size. Expected patient flow was estimated using previous hospital admission records, which indicated an average monthly pediatric admission flow of approximately 359 patients across the pediatric inpatient units. Based on this calculation, a sampling interval of approximately 9 was determined and applied across all pediatric inpatient units. In each ward, the first participant was selected by lottery, and subsequent eligible patients were systematically selected from the admission lists at predetermined sampling intervals until the required number of participants was reached (Figure 1).

### 2.4 Variables and Operational Definitions

The dependent variable was discharge against medical advice (DAMA). Independent variables included age, sex, parental occupation, health insurance status, parental/caregiver educational status, family income, family size, length of hospital stay, patient condition at discharge, diagnosis, residence and distance from hospital, hospital environment, drug availability, and interaction with hospital staff.

DAMA was defined as early discharge from the hospital before completion of treatment, contrary to the recommendation of the treating physician. The length of hospital stay was categorized as < 5 days, 5–10 days, and > 10 days based on clinical relevance, where shorter stays often reflect early discharge or mild conditions, whereas longer stays indicate more severe illness or complications. Additionally, the categorization was informed by the data distribution to ensure meaningful group comparisons. Distance from the hospital was categorized using the median value (33 km) as the cut‐off point.

### 2.5 Data Collection Tools and Procedures

Data were collected using a structured questionnaire through face‐to‐face interviews with parents or caregivers and by reviewing medical charts. Clinical diagnoses were extracted from medical records based on the diagnoses assigned by the treating pediatricians during routine clinical care, and the research team did not modify or reclassify these diagnoses. During hospitalization, discharge outcomes were recorded at the time of discharge for enrolled patients. For patients who discontinued treatment against medical advice, the reason for DAMA was documented by the treating physician at the time of signing the DAMA form as part of routine clinical documentation. These documented reasons were subsequently extracted from medical records by trained data collectors. The research team did not assign, modify, or reclassify DAMA reasons. The questionnaire was adapted from previously published pediatric DAMA studies [13, 16, 17] and modified to suit the local context. It was originally developed in English, translated into Amharic by a bilingual public health expert, and back‐translated into English by an independent translator to ensure consistency. Before data collection, the questionnaire was reviewed by experts in pediatrics and public health to assess clarity, relevance, and contextual appropriateness. The tool assessed sociodemographic characteristics, hospital‐related factors, and staff‐related factors.

### 2.6 Data Quality Control

Data collectors and a supervisor received 2 days of training on the study objectives, tool content, interview techniques, and ethical considerations. The principal investigator checked the collected data daily for completeness and consistency. A pretest was conducted on 5% of the sample to evaluate clarity and feasibility, and the questionnaire was revised based on the feedback before the main data collection.

### 2.7 Data Processing and Analysis

Data were coded and entered into EpiData version 3.1 and exported to SPSS version 26 (IBM Corp., Armonk, NY, USA) for analysis. Descriptive statistics were used to summarize participant characteristics. Continuous variables were summarized using means and standard deviations (SDs) for normally distributed data and medians with interquartile ranges (IQRs) for skewed data, after assessing normality. Categorical variables were summarized using frequencies and percentages.

The prevalence of discharge against medical advice (DAMA) was calculated as the proportion of enrolled pediatric patients who left the hospital against medical advice during the study period.

Binary logistic regression analysis was conducted to assess the association between each independent variable and DAMA. Variables with *p* < 0.25 in the bivariable analysis were entered into the multivariable logistic regression model to control for potential confounding. Adjusted odds ratios (AORs) with 95% confidence intervals (CIs) were reported to quantify the strength and direction of associations. All statistical tests were two‐sided, and statistical significance was declared at *p* < 0.05.

Multicollinearity among independent variables was assessed using variance inflation factors (VIFs), and no significant multicollinearity was observed (VIFs < 10). Model fitness was evaluated using the Hosmer–Lemeshow goodness‐of‐fit test. This study was conducted and reported in accordance with the Strengthening the Reporting of Observational Studies in Epidemiology (STROBE) guidelines for observational studies [19].

### 2.8 Ethical Considerations

Ethical approval was obtained from the Ethical Review Committee of the School of Medicine, University of Gondar (Ref No: SOM/1288/2024). An official permission letter was submitted to the University of Gondar Comprehensive Specialized Hospital. The purpose of the study was explained to parents or caregivers, and informed written consent was obtained before data collection. Participation was voluntary, and confidentiality was strictly maintained by removing personal identifiers.

## 3 Results

### 3.1 Socio‐Demographic Characteristics of Study Participants

A total of 422 pediatric participants were enrolled using systematic sampling during the study period. The age of participants ranged from neonates to children over 10 years, with a median age of 8 months (IQR: 1 week to 3 years), indicating that half of the participants were younger than approximately 8 months. The age distribution was heavily positively skewed, reflecting a high proportion of neonates and infants relative to older children. Of the total participants, 243/422 (57.6%) were males. Neonates were the largest age group (151/422, 35.8%), followed by infants (90/422, 21.3%) and children aged 1–4 years (84/422, 19.9%). More than half of the participants (217/422, 51.4%) did not have health insurance. Most of the patients (190/422, 45%) stayed between 5 and 10 days, whereas 100/422 (24%) stayed less than or equal to 4 days (Table 1).

**Table 1:** Socio‐demographic characteristics of pediatric patients admitted to UGCSH, 2024–2025 (*n* = 422).

Variable	Category	Frequency	Percent (%)
Age	< 28 days	151	35.8
	29 days–11 months	90	21.3
	1–4 years	84	19.9
	5 years and above	97	22.9
Sex	Male	243	57.6
	Female	179	42.4
Residence	Urban	214	50.7
	Rural	208	49.3
Maternal marital status	Married	389	92.2
	Divorced/Widowed	33	7.8
Maternal education	No formal education	201	47.6
	Primary	75	17.8
	Secondary	79	18.7
	Tertiary	67	15.9
Maternal occupation	Housewife	304	72.0
	Government employed	62	14.7
	Self‐employed	56	13.3
Paternal education	No formal education	196	46.4
	Primary	88	20.9
	Secondary	57	13.5
	Tertiary	68	16.1
Paternal occupation	Farmer	213	50.5
	Government employed	102	24.2
	Self‐employed	95	22.5
Health insurance	No	217	51.4
	Yes	205	48.6
Distance from the hospital	< 33 km	212	50.2
	≥ 33 km	210	49.8
Household members	≤ 5	303	71.8
	> 5	119	28.2

### 3.2 Prevalence of Discharge Against Medical Advice

A total of 42 patients out of 422 were discharged against medical advice (DAMA). The prevalence of DAMA was 9.7% (95% CI: 7.1–12.8%) in this study. Of these, 26/42 (62%) were males. Among 97 children aged 5 years and above, 15/97 (15.4%) had DAMA, accounting for 15/42 (35.7%) of all DAMA cases. Of the 151 neonates admitted, 10 (6.6%) left against medical advice.

The most common admission diagnoses among DAMA patients were congenital heart disease (5/42, 11.9%), acute glomerulonephritis (4/42, 9.5%), perinatal asphyxia (4/42, 9.5%), sepsis (3/42, 7.1%), and leukemia (3/42, 7.1%). Among neonates who left against medical advice, perinatal asphyxia was the most frequent diagnosis (6/10, 40.0%), followed by neonatal jaundice (2/10, 20%). DAMA was most frequent in the pediatric inpatient ward (15/42, 36.5%), followed by the pediatric emergency (10/42, 24.3%), and the NICU (10/42, 24.3%).

### 3.3 Reasons for Discharge Against Medical Advice

**Figure 2:**
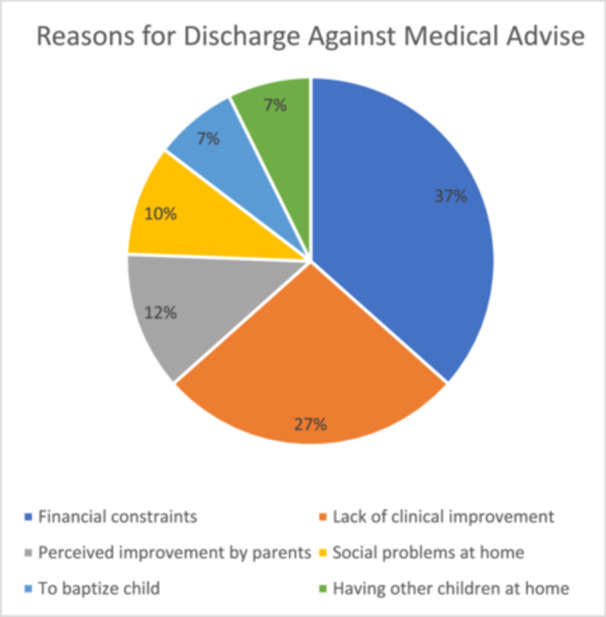
Reported reasons for discharge against medical advice (DAMA) among pediatric patients admitted to the University of Gondar Comprehensive Specialized Hospital, Northwest Ethiopia, 2024–2025 (*n* = 42).

The most common reasons given for DAMA were financial constraints (15/42, 35.7%), followed by lack of clinical improvement (11/42, 26%), and perceived improvement by parents (5/42, 11.4%) (Figure 2). Among neonates, the most common reason for DAMA was lack of clinical improvement (5/10, 50%), followed by social problems at home and financial constraints (2/10, 20% each). Among the 42 patients discharged against medical advice, 38 (90.5%) were taken home, 3 (7.1%) were taken to private clinics, and the remaining 1 (2.4%) was taken to a holy water site.

### 3.4 Factors Associated With Discharge Against Medical Advice

In the bivariable logistic regression analysis, parental education, parental occupation, health insurance status, monthly family income, duration of hospitalization, and residence met the inclusion criteria (*p* < 0.25) and were entered into the multivariable logistic regression model.

In the multivariable logistic regression model, maternal education remained the only independent predictor of DAMA. Children of mothers with secondary education had 68% (AOR = 0.32; 95% CI: 0.11–0.99; *p* = 0.04), and those whose mothers had tertiary education had 81% (AOR = 0.19; 95% CI: 0.04–0.84; *p* = 0.03) lower odds of DAMA compared to children of mothers with no formal education.

Age of the child, health insurance status, and maternal marital status were not significantly associated with DAMA in the multivariable model. Although children aged 1–4 years and ≥ 5 years showed higher odds of DAMA compared with infants, the associations were not statistically significant (AOR = 2.17; 95% CI: 0.83–5.71 and AOR = 1.94; 95% CI: 0.79–4.75), respectively. Similarly, having health insurance (AOR = 0.73; 95% CI: 0.37–1.41) and being from divorced or widowed mothers (AOR = 0.22; 95% CI: 0.03–1.67) were not significantly associated with DAMA (Table 2).

**Table 2:** Bivariable and multivariable logistic regression of factors associated with discharge against medical advice (DAMA) among pediatric patients, UGCSH, 2024–2025.

Variable	Category	COR (95% CI)	*p*‐value	AOR (95% CI)	*p*‐value
Maternal Education	No education	Reference		Reference	
	Primary	0.73 (0.33–1.64)	0.45	0.82 (0.36–1.90)	0.64
	Secondary	0.31 (0.11–0.92)	0.03	0.32 (0.11–0.99)	**0.04**
	Tertiary	0.19 (0.04–0.80)	0.02	0.19 (0.04–0.84)	**0.03**
Age of the child	Neonate	Reference		Reference	
	Post‐neonate	1.19 (0.44–3.24)	0.73	0.98 (0.35–2.73)	0.96
	Under‐five (1‐4 yrs)	1.91 (0.76–4.78)	0.17	2.17 (0.83–5.71)	0.15
	5 years and above	2.58 (1.11–6.01)	0.02	1.94 (0.79–4.75)	0.15
Insurance	No	Reference		Reference	
	Yes	0.99 (0.52–1.87)	0.09	0.73 (0.37–1.41)	0.34
Maternal Marital Status	With partner	Reference		Reference	
	Without partner	0.27 (0.04–1.99)	0.19	0.22 (0.03–1.67)	0.14

## 4 Discussion

The prevalence of discharge against medical advice (DAMA) in this study was 9.7% (95% CI: 7.1%–12.8%). This finding is comparable to reports from Bouake, Côte d'Ivoire (12%) and Bayelsa State, Nigeria (7.5%) [20, 21]. However, the prevalence observed in this study was higher than that reported from Benin City, Nigeria (6.3%) and India (5.2%) [22, 23]. These differences may be partly explained by variations in study design, study populations, healthcare settings, hospital admission profiles, disease severity, referral pathways, and discharge practices. For example, the study from Benin City included only hospitalized children younger than 5 years, whereas the present study included pediatric patients younger than 18 years, potentially capturing a broader spectrum of illnesses and caregiver‐related factors associated with DAMA. The prevalence was considerably higher than that reported from Oman (0.32%) [24], which may reflect differences in healthcare financing, universal health coverage, socioeconomic conditions, and healthcare delivery systems compared with low‐ and middle‐income settings.

In the present study, Maternal education remained the only variable significantly associated with DAMA in the multivariable analysis. Children whose mothers had secondary or tertiary education were significantly less likely to be discharged against medical advice compared to those whose mothers had no formal education. This suggests that maternal education may be associated with healthcare decision‐making for children. Because caregiver decisions are influenced by multiple social, cultural, and health‐system factors, additional unmeasured variables may also contribute to DAMA.

Several mechanisms may explain this association. Educated mothers are more likely to have better health literacy, enabling them to understand the severity of illness, the importance of completing treatment, and the potential risks of premature discharge. Higher educational attainment may also improve communication and trust between caregivers and healthcare providers, enhancing adherence to medical advice [25, 26]. Additionally, maternal education is often linked with higher socioeconomic status, greater autonomy in household decision‐making, and stronger confidence in formal healthcare systems, collectively reducing the likelihood of DAMA. These findings align with previous studies in similar settings [10, 12, 25], where low parental education was associated with higher DAMA rates.

In this study, DAMA was most prevalent among children aged 5–10 years (17%), while neonates had a lower prevalence (6.6%). This pattern is consistent with some previous studies but contrasts with reports from Benin City, Nigeria, where DAMA was higher among infants and neonates [23]. The lower prevalence in neonates in our study may be attributed to partial coverage of newborn care costs, which reduces financial barriers for parents.

We observed no significant association between age, sex, or residence and DAMA, differing from findings reported in Iran [27]. However, bivariate analysis indicated associations between DAMA and parental occupation, health insurance status, family income, duration of hospitalization, and residence inside or outside Gondar, consistent with previous literature.

Among non‐neonatal patients, congenital heart disease, acute glomerulonephritis, and leukemia were the most frequent diagnoses among DAMA cases, reflecting the local disease burden. In neonates, perinatal asphyxia, neonatal jaundice, and respiratory distress were the most common conditions among DAMA cases, consistent with patterns reported in Nigerian studies [23].

Financial constraints were the most frequently cited reason for DAMA, emphasizing the critical role of economic factors in healthcare utilization. These findings are consistent with systematic evidence highlighting caregiver‐related, financial, and health‐system determinants of DAMA across diverse settings [28]. Lack of clinical improvement and perceived early recovery were also commonly reported reasons, particularly among neonates [5, 10, 15]. Occasional unavailability of medications within the hospital, which required parents to purchase from private pharmacies, may have further contributed to premature discharge.

## 5 Conclusions

This study found that 9.7% of pediatric patients at the University of Gondar Comprehensive Specialized Hospital were discharged against medical advice. Maternal education was the only independent predictor of DAMA, with children of mothers with secondary or tertiary education significantly less likely to leave prematurely. Financial constraints and perceived lack of clinical improvement were the leading reasons for DAMA, suggesting that caregiver knowledge and understanding of illness may be important factors in hospital discharge decisions.

To reduce DAMA and improve pediatric health outcomes, we recommend strengthening caregiver education on the importance of completing treatment, particularly for children with severe or chronic illnesses. Improving hospital services, including consistent medication availability and supportive care, may also help parents feel more confident in continuing treatment.

### 5.1 Strengths and Limitations

The prospective enrollment approach allowed participants to be identified before the occurrence of DAMA events. The findings provide important insights for policymakers and hospital administrators to address DAMA and improve pediatric care. The study was conducted at a single tertiary teaching hospital; therefore, the findings may not be generalizable to other hospitals or regions. Reasons for DAMA were obtained from routine clinical documentation and caregiver explanations recorded during the discharge process; therefore, incomplete documentation or reporting bias cannot be completely excluded. The study also did not classify pediatric illnesses as acute or chronic, which may have limited exploration of potential differences in DAMA across disease categories. Clinical diagnoses were based on routine clinical assessments by treating pediatricians and were not independently standardized or verified by the research team; therefore, variability in diagnostic practices among clinicians could not be completely excluded.

## Author Contributions

**Tesfaye Shumet Mekonnen:** writing – original draft, funding acquisition, conceptualization, investigation, methodology, validation, visualization, writing – review and editing, software; formal analysis, project administration, data curation, supervision, resources. **Demewoz Fikir:** conceptualization, investigation, funding acquisition, methodologym validation, visualization, software, project administration, formal analysis, resources, supervision, writing – original draft.

## Funding

The authors have nothing to report.

## Conflicts of Interest

The authors declare no conflicts of interest.

## Author Responsibility Statement

All authors have read and approved the final version of the manuscript. **Tesfaye Shumet Mekonnen** had full access to all study data and takes complete responsibility for the integrity of the data and the accuracy of the analysis.

## Transparency Statement

Tesfaye Shumet Mekonnen affirms that this manuscript is an honest, accurate, and transparent account of the study being reported; that no important aspects of the study have been omitted; and that any discrepancies from the study as planned have been explained.

## Data Availability

The data that support the findings of this study are available from the corresponding author upon reasonable request.
